# 3D Printing of Continuous Natural Fiber-Reinforced Thermoset Composites

**DOI:** 10.3390/polym18172152

**Published:** 2026-09-03

**Authors:** Md Zahirul Islam, Swetha Manoharan, Gavin Kahn, Prashant Lakhemaru, Rubayed Razib, Luke Gibbon, Rachel Workin, Chad A. Ulven

**Affiliations:** 1 Department of Mechanical Engineering, North Dakota State University, Fargo, ND 58102, USA; swetha.manoharan@ndsu.edu (S.M.); gavin.kahn@ndsu.edu (G.K.); prashant.lakhemaru@ndsu.edu (P.L.); rubayed.razib@ndsu.edu (R.R.); luke.r.gibbon@ndsu.edu (L.G.); rachel.tharalson.2@ndsu.edu (R.W.); 2 Department of Mechanical and Civil Engineering, Minnesota State University, Mankato, MN 56001, USA; mdzahirul.islam@mnsu.edu

**Keywords:** hemp fiber, 3D printing, flax fiber, thermoset resin, mechanical characterization

## Abstract

Natural fiber-reinforced composites improve environmental sustainability, and their use is increasing as environmental awareness grows. Natural fibers, such as hemp and flax, are renewable resources. They offer several advantages, including abundant availability, low cost, and high biodegradability. Three-dimensional printing has introduced a novel approach for advancing natural fiber-based composites. However, there is a significant lack of research on the 3D printing of continuous hemp and flax fiber-reinforced thermoset composites. This research investigates the fabrication of hemp and flax fiber-reinforced thermoset composites using a UV-light-assisted 3D printing process. Both printed composites achieved a fiber volume fraction of approximately 13%. The tensile strength of hemp and flax fiber-reinforced composites was found to be 32.3 ± 3.5 MPa and 61.1 ± 4.2 MPa, respectively. Moreover, the flexural strengths of hemp and flax fiber-reinforced composites were 81.8 ± 13.5 MPa and 85.3 ± 23.1 MPa, respectively. Thus, this study may open research pathways for utilizing natural fiber-reinforced thermoset composites with improved biodegradability and renewability.

## 1. Introduction

Continuous fiber-reinforced polymer (CFRP) composites are known for their high strength-to-weight ratio, making them widely used in the mechanical, construction, aerospace, and automobile industries [1]. They also offer excellent design flexibility. During the development of these composites, low-cost manufacturing technologies have been an important topic of research.

Traditional manufacturing of fiber-reinforced composites is costly, labor-intensive, inflexible, and time-consuming. It requires customized rigid molds. In contrast, additive manufacturing offers greater design flexibility along with a rapid and cost-effective fabrication process [2]. Moreover, additive manufacturing saves materials.

The 3D printed CFRP composites have demonstrated enhanced mechanical properties. Most studies have focused on the 3D printing of synthetic fiber-reinforced composites [3]. Commonly used synthetic fibers include carbon fiber, glass fiber, and aramid fiber. Continuous carbon fiber (synthetic) has been widely utilized for 3D printing with thermoset resin. They were commonly 3D printed using an extrusion-based direct ink write (DIW) printing process or UV-light-assisted printing process. UV-light-assisted processes show promise in printing with low viscosity thermoset resin; however, the DIW printing process requires very highly viscous thermoset resin.

Carbon fiber-reinforced CFRP composites show mechanical properties comparable to those of metals. However, A major disadvantage of these composites is their limited material availability, being non-renewable, and associated environmental hazards. Meeting environmental protection goals is challenging when using synthetic fiber-reinforced composites [4].

Under these circumstances, 3D printing of natural fiber-reinforced composites has become the focus of attention of industries and academia due to their sustainability and low-cost. In recent years, an increasing number of agricultural crops, such as jute, bamboo, pineapple leaf, hemp, and flax, have been used as reinforcing phases in composites to address serious environmental concerns [5]. Plant-based fibers are being increasingly adopted for composite reinforcement.

Natural fibers are increasingly used in additive manufacturing due to their biodegradability, renewability, sustainability, and eco-friendly nature. These make them valuable contributors to the circular economy [6]. The fabrication and utilization of natural fiber-reinforced composites improve environmental friendliness and sustainability. Therefore, the study of 3D printing of continuous natural fibers has received more attention in recent years.

Numerous studies have been conducted on 3D printing with natural fibers using thermoplastic matrix materials. Matsuzaki et al. [7] first demonstrated the 3D printing of continuous jute yarn-reinforced thermoplastic composites using in-nozzle impregnation. Polylactic acid (PLA) was used as a matrix material. The resulting composites exhibited a tensile strength of 57 MPa and a tensile modulus of 5.11 GPa.

Santos et al. [8] developed a 3D printing process for continuous vegetable yarn-reinforced thermoplastic composites, using PLA as matrix material. They observed that the reinforced samples achieved a 28.8% increase in tensile strength and a 28.9% increase in modulus compared to the unreinforced matrix. Furthermore, Cheng et al. [9] conducted the mechanical and interfacial analysis of 3D-printed continuous ramie fiber-reinforced thermoplastic bio-composites, using PLA as the matrix material. They investigated the effects of various printing parameters and reported a maximum tensile strength of 86 MPa.

Terekhina et al. [10] 3D printed continuous flax fiber-reinforced thermoplastic composites using polyamide as the matrix material. They employed an in-nozzle impregnation-based printing process and investigated the effects of various infill patterns. The maximum tensile strength achieved was approximately 82 MPa. Moreover, Duigou et al. [11] performed 3D printing of continuous flax fiber-reinforced composites using PLA as the matrix material. They achieved a tensile strength of 253 MPa and a modulus of 3.5 GPa.

Furthermore, plant fibers possess abundant hydrophilic hydroxyl groups that are inherently incompatible with hydrophobic resins. The interfacial compatibility between fiber and matrix is widely recognized as a critical factor governing the mechanical performance of fiber-reinforced composites. Long et al. [12] treated the surface of the flax fiber yarn with a silane coupling agent to improve wettability and interfacial performance of 3D printed flax/PLA composites.

Moreover, the twisted structure of natural fiber yarns restricts polymer impregnation, resulting in a lower fiber volume fraction in 3D-printed composites. Yang et al. [13] demonstrated a method for untwisting flax fiber yarn using rotational vibration to improve impregnation with PLA resin.

Natural fiber composites have recently been used to fabricate honeycomb cores for sandwich panels, offering advantages such as vibration control, improved energy absorption, and enhanced impact performance. Antony et al. [14] demonstrated the 3D printing of a honeycomb sandwich structure using hemp fiber-reinforced PLA filament. The aim of their study was to minimize plastic usage without compromising mechanical properties, thereby reducing the overall production cost of the final components. Additionally, Zhang et al. [15] demonstrated the 3D printing of continuous flax fiber-reinforced PLA composites using a 5-axis printer. They 3D-printed a leaf spring and a shoe cap using this technology.

Thermosetting polymers have gained significant importance in recent decades owing to their superior thermal stability, chemical resistance, and mechanical strength. Unlike thermoplastics, once cured, thermosets cannot be reshaped because their crosslinked polymer network is irreversible. This characteristic makes them particularly suitable for manufacturing permanent components and large, structurally robust parts [16].

However, to the best of the author’s knowledge, no research has been conducted on the 3D printing of continuous natural fibers using a liquid thermoset resin system. The unique contribution of this preliminary research is to demonstrate the capability to 3D-print continuous hemp and flax fiber-reinforced thermoset composites using a UV-light-assisted printing process. Furthermore, this study also includes the physical and mechanical characterization of printed composites.

## 2. Materials and Methods

### 2.1. Materials

**Figure 1:**
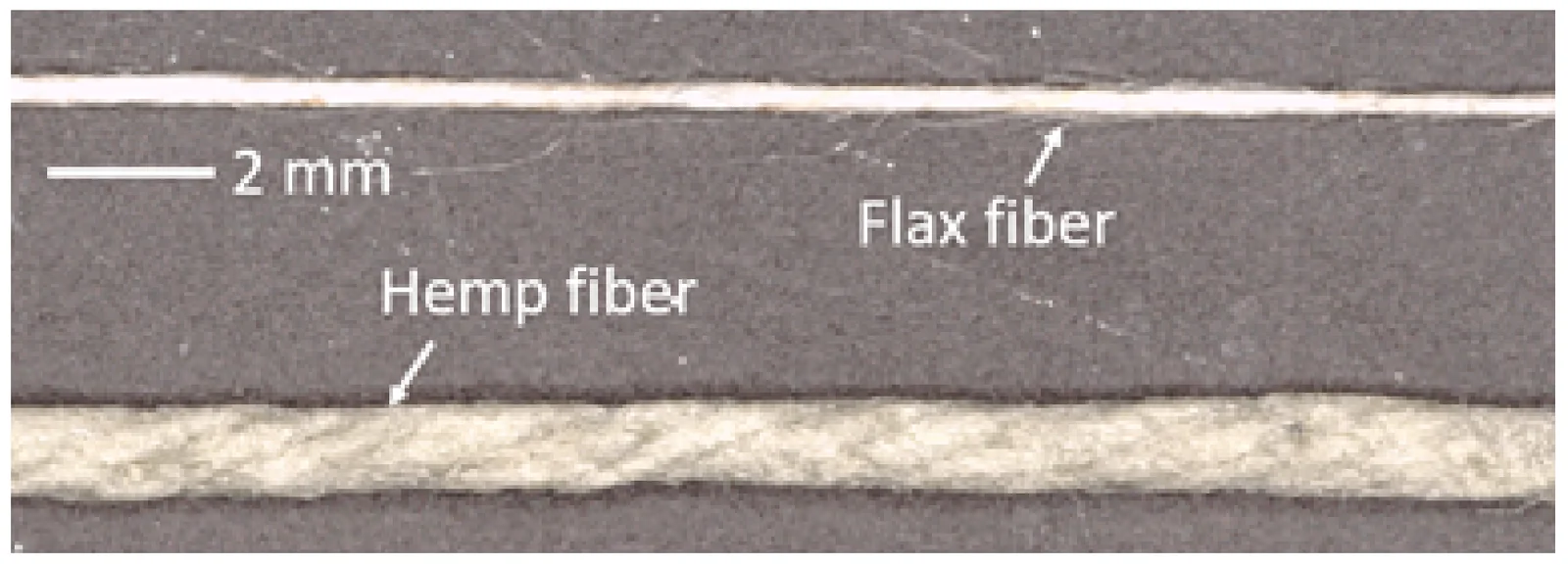
Continuous natural fiber used in this study (hemp and flax).

To conduct the 3D printing, continuous hemp fiber was purchased from Natural Source Hemp (Qingdao, Shandong, China). Figure 1 shows the continuous hemp fiber used in this study. The linear mass density of the commercially purchased hemp fiber was 459 tex, and its volumetric density was 1490 kg/m^3^. The fiber was twisted, with an average diameter of 1 mm.

In addition, continuous flax fiber yarn (BComp amlitex, Fribourg, Switzerland) was used as the reinforcing element. Figure 1 shows the continuous flax fiber used. The linear mass density of the flax fiber was 95 tex, and its volumetric density was 1330 kg/m^3^. The fiber was twisted in style, with a diameter of 0.3 mm. Figure 1 clearly shows that the hemp fiber was more twisted than the flax fiber. No chemical treatment or surface modification was applied to the hemp and flax fibers prior to printing. Both the hemp and flax fibers were used in their as-received condition.

The liquid thermoset resin used for 3D printing was Bisphenol-A epoxy acrylate (BEA), obtained from Covestro (Pittsburgh, PA, USA). This BEA is commonly used in energy-curable systems (ultraviolet/electron beam). The commercial name of this resin is Agisyn 1010-A60 (Leverkusen, Germany). To make the resin curable under 405 nm ultraviolet light, a photo-initiator (2,4,6-trimethylbenzoyl) phosphine oxide (97%) was mixed with the resin. The photo-initiator was obtained from Sigma-Aldrich (St. Louis, MO, USA).

To make the resin thermally curable, a thermal initiator (2% K-PURE CXC-1612, Sigma-Aldrich) was also mixed with the resin. The resin was combined with the photo-initiator and thermal initiator in a mass ratio of 100:2:2. To ensure complete mixing, the mixture was placed in a speed mixer at 2000 rpm for 2 min. The mixed resin was left overnight to degas. The Newtonian viscosity of the mixed resin was 2.77 Pa·s.

### 2.2. Printing Process

**Figure 2:**
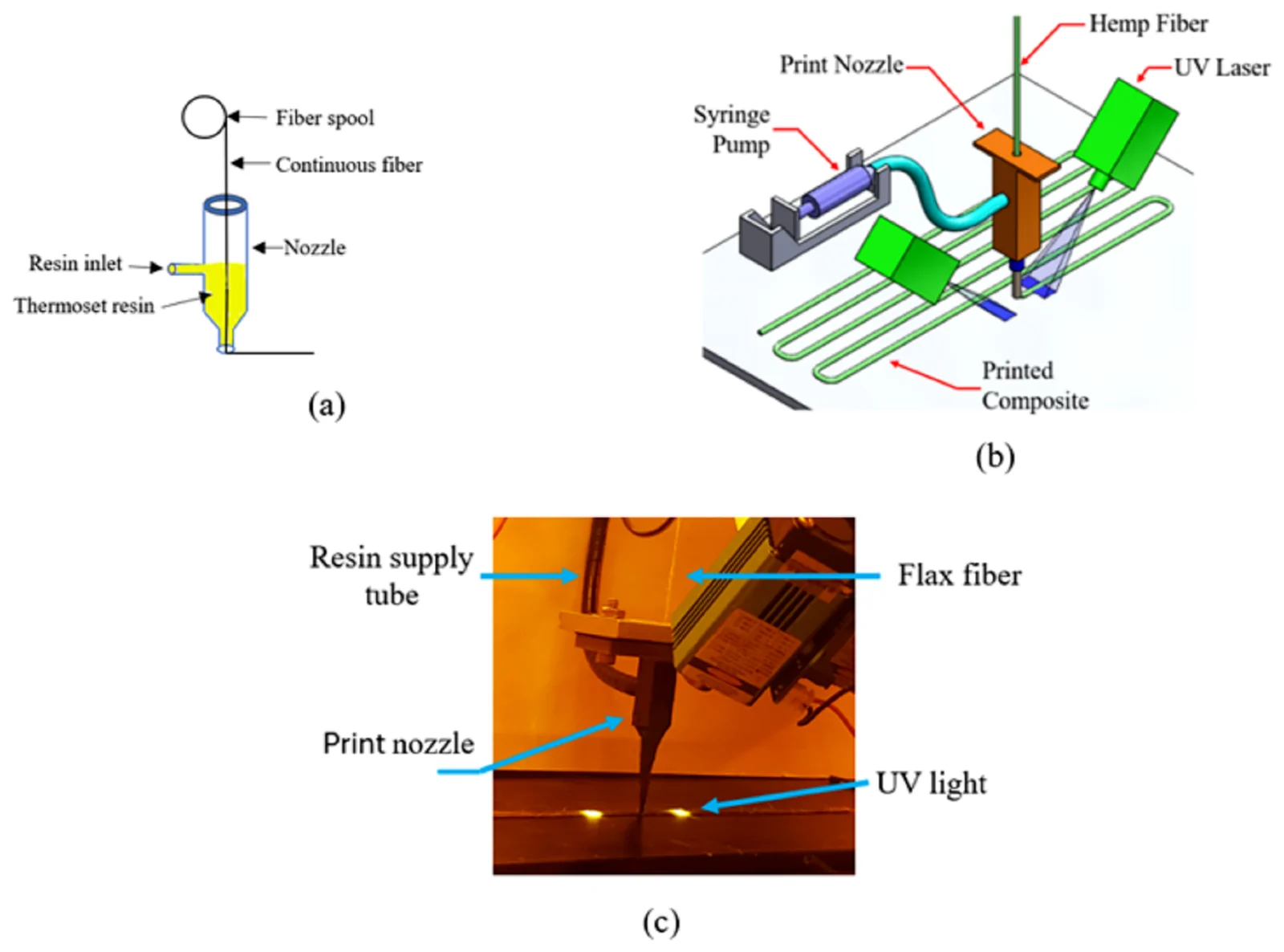
(**a**) Custom print head. (**b**) Schematic of the printing process. (**c**) Real-life printing process.

Figure 2 shows the printing process used to fabricate continuous hemp and flax fiber composite specimens. A commercial X- and Y-axis movable gantry was modified to build the custom 3D printer. The motion of the gantry was controlled using motor controllers and Arduino IDE. As shown in Figure 2a, a custom print nozzle was designed to feed continuous fiber (hemp or flax) and thermoset resin simultaneously. A syringe tip was attached to the end of the nozzle. Continuous fiber was fed through the top of the nozzle. The thermoset resin was introduced from the side using a syringe pump. The resin feed rate was 0.363 mL/min for hemp fiber and 0.121 mL/min for flax fiber. The continuous fiber was impregnated with liquid resin inside the print nozzle. Figure 2b,c shows the schematic and real-life printing process of the study.

The continuous fiber was pulled from the nozzle due to the motion of the gantry. A UV light source was used to solidify the liquid thermoset resin on the print bed. Two UV lasers, each with a wavelength of 405 nm and a power of 0.8 W, were focused at a specific distance of approximately 20 mm in front of and behind the nozzle tip. One laser cured the resin as the print nozzle moved from left to right, while the other was used as the print nozzle moved from right to left.

As shown in Figure 2b, the unidirectional specimens were 3D printed using the back-and-forth motion of the print nozzle. The raster gap between two consecutive fiber lines was 1.25 mm for hemp fiber and 0.67 mm for flax fiber. The layer height was approximately 1.7 mm for hemp fiber composites and 1 mm for flax fiber composites. Each layer contained ten fiber lines in the hemp fiber composites, whereas each layer had fourteen lines per layer. The print speed was set to 120 mm/min for the hemp fiber-reinforced composites, while it was set to 210 mm/min for the flax fiber-reinforced specimens. For mechanical characterization, two-layer specimens were 3D printed for the hemp fiber composites (average thickness = 3.4 mm), whereas the flax fiber-reinforced composites consisted of three layers (average thickness = 3 mm).

Natural fibers generally allow greater ultraviolet (UV) light transmission than carbon fibers. The carbon fibers are essentially opaque to UV radiation. Consequently, natural fiber reinforcements present less of a barrier to UV-induced curing of photopolymerizable thermoset resins [17]. Consequently, UV exposure sufficiently solidified the resin to retain the shape of the printed fiber on the bed. Thermal curing was then required to achieve complete solidification. Both hemp and flax fiber-reinforced printed specimens were thermally cured at 150 °C for 5 h. Atik et al. [18] used a similar resin in their study and performed differential scanning calorimetry (DSC) analysis. The DSC results showed no significant thermal peaks for the cured specimens, indicating the absence of residual curing reactions. Therefore, the degree of cure achieved after post-curing can be considered sufficient.

### 2.3. Print Quality

**Figure 3:**
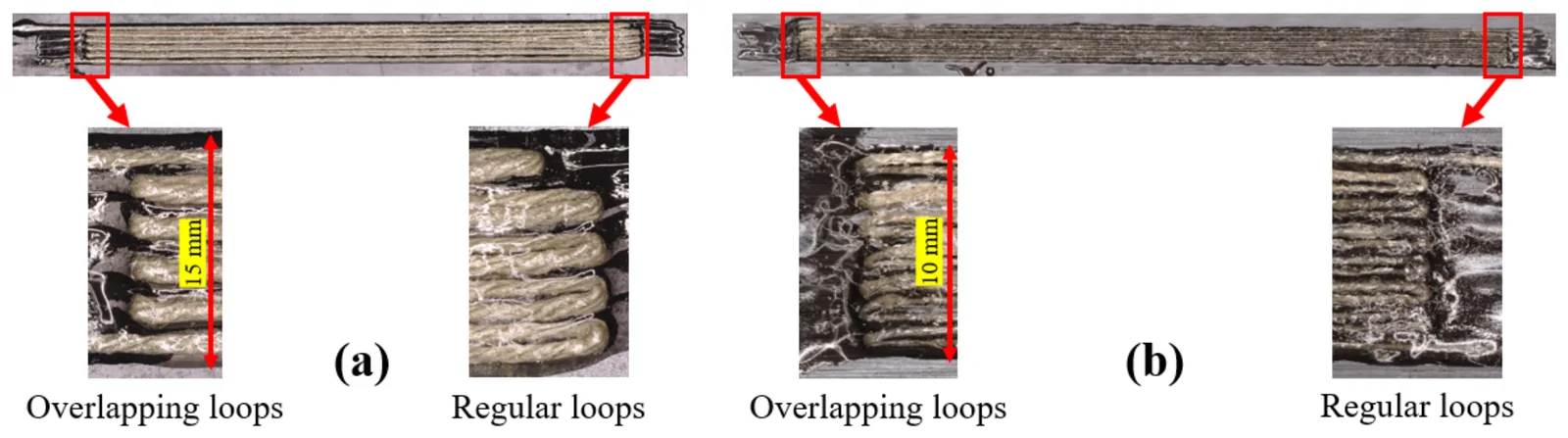
Overlapping loops and regular loops: (**a**) hemp fiber composites, (**b**) flax fiber composites.

Figure 3a shows a single layer of the 3D-printed continuous hemp fiber-reinforced thermoset composite, while Figure 3b shows that for flax fiber-reinforced composites. The print nozzle moved back and forth to create a unidirectional rectangular specimen. Fiber orientation is responsible for the macroscopic strength, stiffness, and structural behavior of composite material [19]. Enlarged views of the specimen’s ends show how the continuous fiber naturally folded during the deposition of the adjacent parallel line. This is clear from Figure 3 that the loops at one side were regular in shape, whereas the loops on the other side overlapped one another. Similar observations of regular and overlapping loops were reported by Duigou et al. [11].

**Figure 4:**
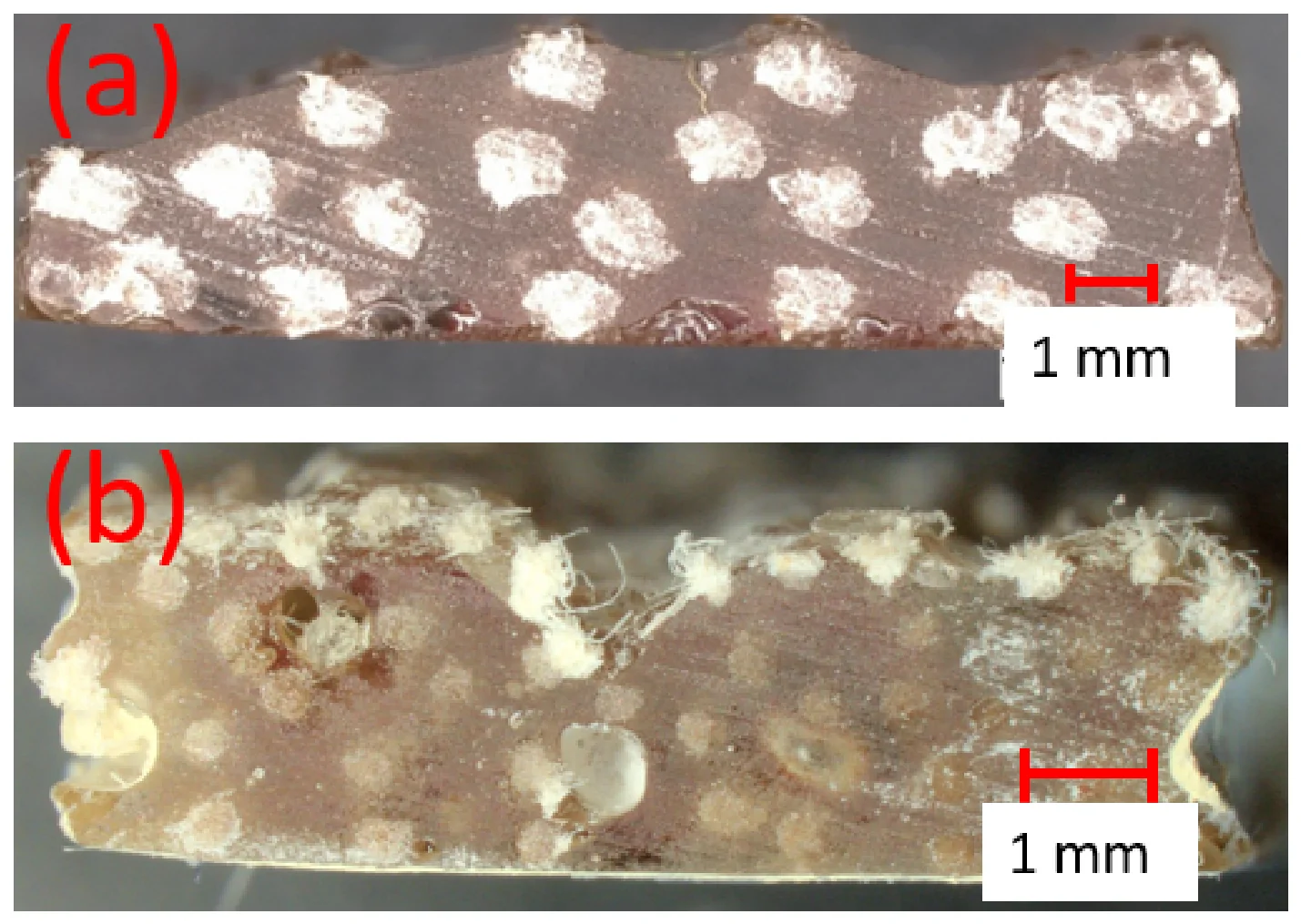
Cross-sectional image of the 3D printed composites: (**a**) hemp fiber composites, (**b**) flax fiber composites.

Figure 4 shows the cross-section of the 3D-printed composite. The printed specimens were sectioned transversely and observed under the optical microscope (Keyence, VHX-6000, Osaka, Japan). Figure 4 further shows that the thickness of the printed specimen was non-uniform across its width. This non-uniformity resulted from the formation of overlapping loops during printing. The hemp fiber-reinforced composite consists of two fiber layers, whereas the flax fiber-reinforced composite consists of three fiber layers. However, in the cross-section shown in Figure 4, the fibers appear to be arranged in multiple layers due to the formation of overlapping loops during each layer’s printing. The overlapped fibers are positioned at a greater height than the regularly deposited fibers.

### 2.4. Fiber Volume Fraction and Density Measurement

The fiber volume fraction of the printed composites was determined using an analytical approach. In this method, the total fiber length ($L_{f}$, in mm) was obtained by multiplying the specimen length (*l*) by the total number of fibers in the cross-section (*n*). This total fiber length was then multiplied by the tex value to compute the total fiber mass ($m_{f}$). The mass of the fiber can be expressed mathematically as shown in Equation (1) [20]:

(1) $$m_{f}=\frac{L_{f}\times tex}{10^{6}}$$

where tex is the linear mass density of the fiber. The fiber volume ($v_{f}$) was calculated by dividing the measured fiber mass by the fiber’s density (ρ_f_). Likewise, the matrix volume ($v_{m}$) was determined by dividing the mass of the matrix ($m_{m}$) by its density (ρ_m_). The overall fiber volume fraction was then computed as the ratio of the fiber volume to the total volume of the composite, i.e., $v_{f}/(v_{f}+v_{m})$.

Furthermore, the density of the 3D-printed composite was measured using Archimedes’ principle [21]. A Mettler Toledo Density Kit (from Fisher Scientific, Waltham, MA, USA) was used for this analysis. Prior to testing, the specimens were cleaned and dried to remove any surface contaminants and moisture. Each specimen was first weighed in air to obtain its dry mass. The specimen was then suspended in an immersion fluid, and its apparent mass while fully submerged was recorded. Acetone was selected as the immersion fluid instead of water because the natural fiber-reinforced composites tended to float in water, preventing complete submersion and reducing measurement accuracy. The density of acetone at room temperature (784 kg/m^3^) was used in the calculations. Based on Archimedes’ principle, the difference between the specimen mass measured in air and its apparent mass in acetone corresponds to the buoyant force, which is equal to the weight of the displaced fluid. The composite density was therefore calculated using Equation (2):

(2) $$\rho_{c}=\frac{m_{\mathrm{air}}}{m_{\mathrm{air}}-m_{\mathrm{acetone}}}\times \rho_{\mathrm{acetone}}$$

where ρ_c_ is the density of the composite, m_air_ is the specimen mass measured in air, m_acetone_ is the apparent mass of the specimen while immersed in acetone, and ρ_acetone_ is the density of acetone at room temperature. Each specimen was measured five times, and the average value was reported.

### 2.5. Surface Analysis

**Figure 5:**
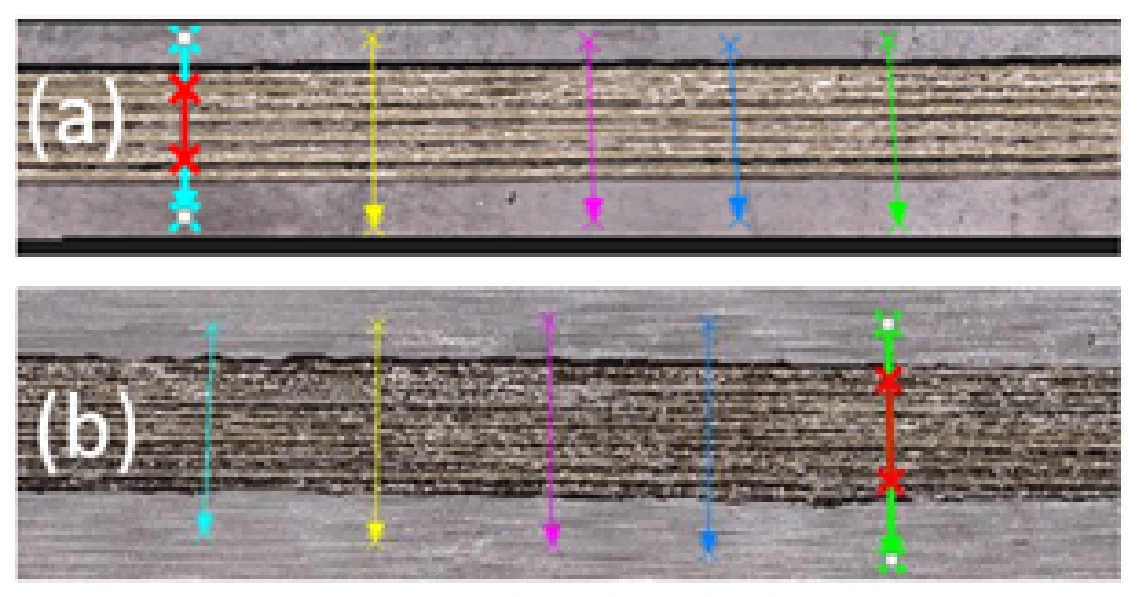
Surface roughness measurement (**a**) for hemp fiber, (**b**) for flax fiber.

A single layer of continuous hemp and flax fiber was 3D printed to evaluate the line roughness (R_a_). Line roughness of the printed layer was measured using an optical profilometer (Keyence VR-6200, Osaka, Japan). As shown in Figure 5, the line roughness was measured along several lines on the printed layer in the transverse direction to the fiber. A total of five lines were used to calculate the average line roughness. Furthermore, the optical profilometer was used to capture the surface profile in the transverse direction of the print.

### 2.6. Tensile Test of Hemp and Flax Fiber

**Figure 6:**
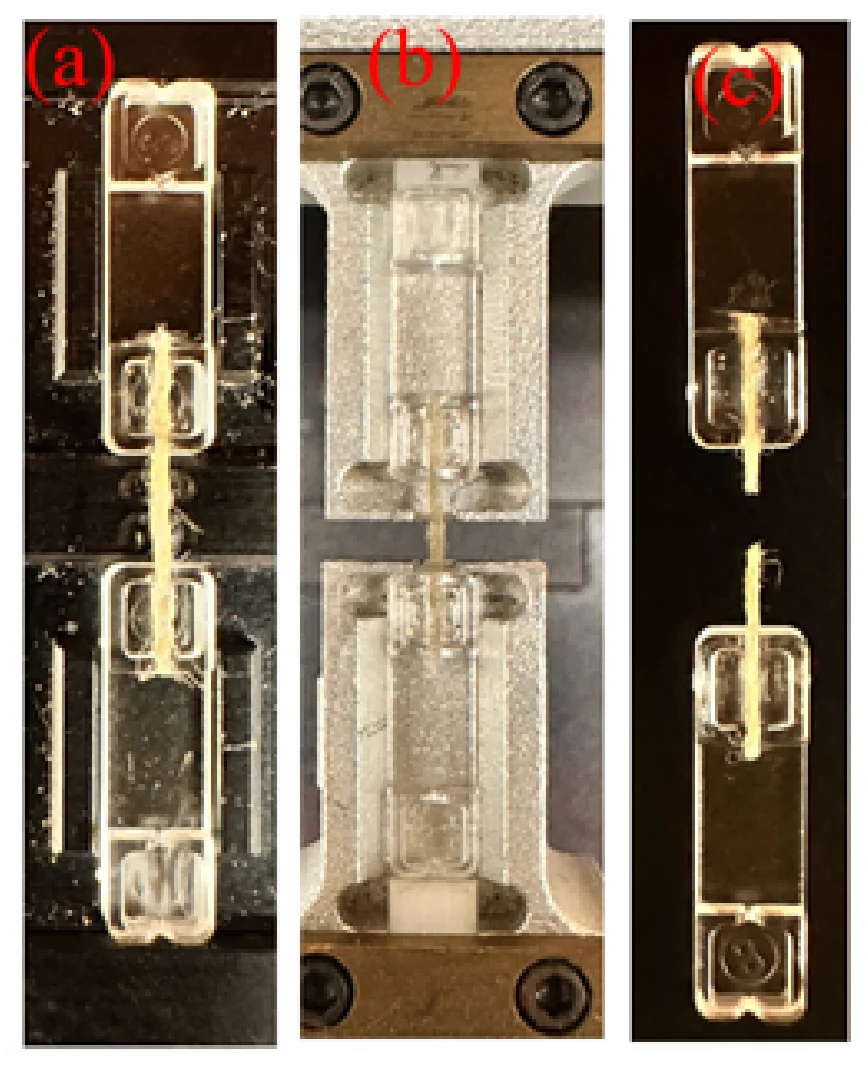
Tensile test of individual hemp fiber: (**a**) fiber loaded in the cassette, (**b**) fiber cassette on load cell, (**c**) tensile tested fiber.

The tensile test of individual hemp and flax fibers was performed using a Dia-Stron fiber testing machine (capacity: 20 N). Figure 6a shows the fiber loaded on the cassette using glue. Figure 6b shows the fiber-loaded cassette on the tensile testing load cell. Gauge length was 4.3 mm. Test was conducted at a rate of 12 mm/min. Figure 6c shows the failed fiber after the tensile test.

### 2.7. Tensile Test of Printed Composites

The tensile test of 3D printed composites was performed using an MTS electromagnetic load frame following ASTM D3039 [22] standard. Five specimens were tested to ensure repeatability. To perform the tensile test, two-layer unidirectional specimens were 3D printed for hemp fiber composites, while three-layer specimens were 3D printed for flax fiber composites. Tabs were inserted at both ends to ensure proper gripping and load transfer. As shown in Figure 4, the thickness of the specimens was not uniform. Hence, the cross-sectional area of the specimen was measured using an optical microscope. The tensile strength was measured by dividing the force by the cross-sectional area measured using a microscope. The test was conducted at a crosshead rate of 1 mm/min.

**Figure 7:**
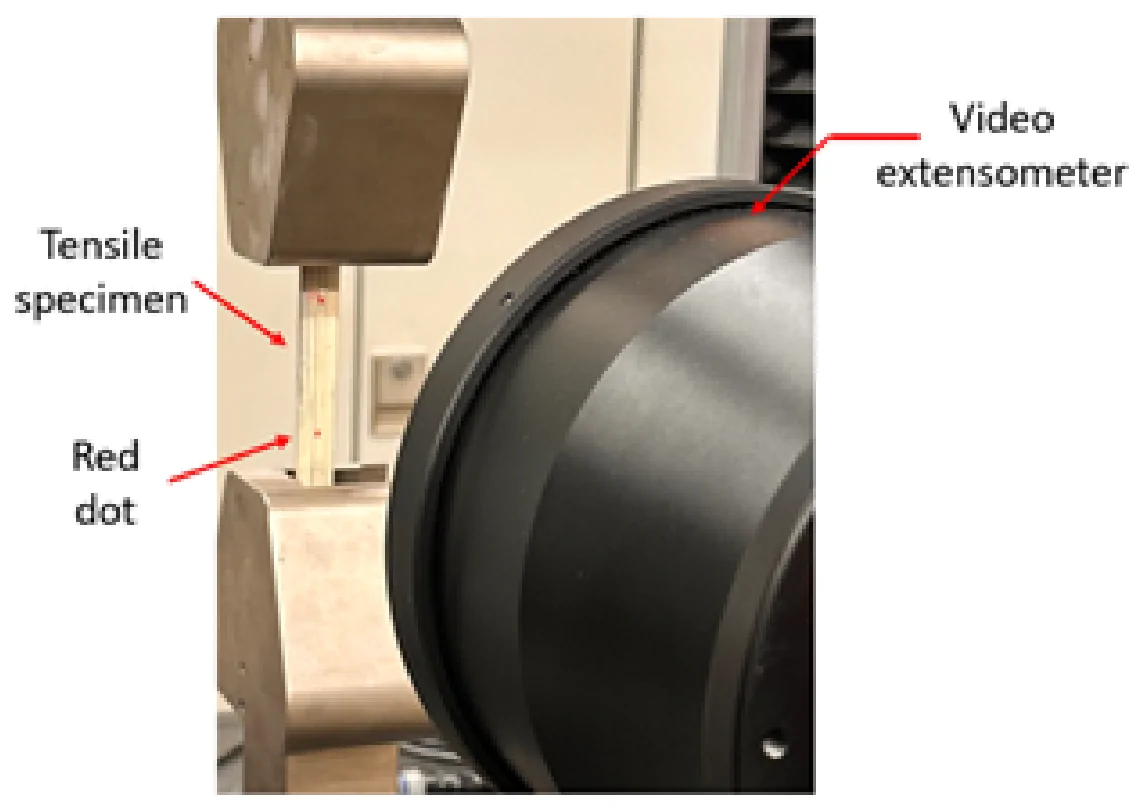
Tensile test of 3D printed composites with video extensometer.

An MTS video extensometer (AVX-205) was used to measure strain during the tensile test (MTS, Eden Prairie, MN, USA). Two dots were placed on the specimen along the longitudinal direction. The video extensometer tracked the displacement of these two points to measure the strain. The experimental tensile test setup with the video extensometer is shown in Figure 7.

### 2.8. Flexural Test of Printed Composites

The flexural test was performed using a three-point bending configuration, following the ASTM D7264 [23] standard. A three-point bending configuration was used due to its simplicity and ease of implementation. Similar to the tensile test, two-layer composite specimens were 3D printed for the flexural test of hemp fiber-reinforced composites, while three-layer specimens were used for flax fiber-reinforced composites. A total of five specimens were tested for each composite. The average width of the specimens was 15.1 mm for hemp fiber-reinforced composites and 10.5 mm for flax fiber-reinforced composites. However, as shown in Figure 4, the thickness of the specimens was not uniform across the cross-section. Therefore, the cross-sectional area of the specimen was measured using an optical microscope. The modified flexural stress (σ) and strain (ϵ) formulas, shown in Equations (3) and (4), were used. Where, P is the applied force (N), L is span (mm), b is width (mm), A is cross-sectional area (mm^2^), and δ is the mid-point deflection (mm) of the specimen.

(3) $$\sigma =\frac{3PLb}{2A^{2}}$$

(4) $$\epsilon =\frac{6\delta A}{L^{2}b}$$

**Figure 8:**
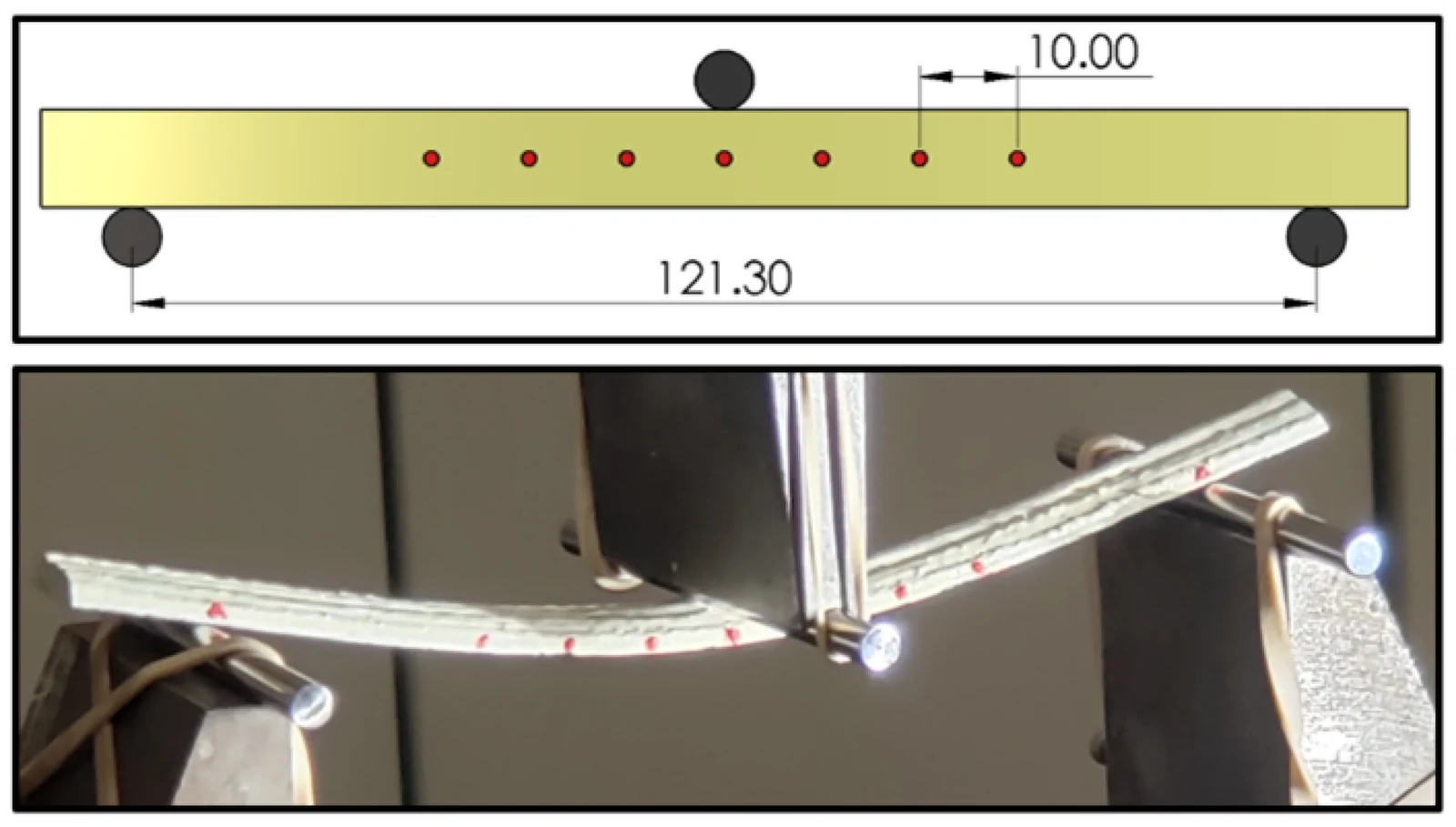
(**Top**) Schematic and (**Bottom**) real-life image of the flexural test using a video extensometer.

The diameter of the loading roller and the support rollers was 4.75 mm. During the test, a span-to-thickness ratio of 32:1 was used. The test rate was set at 1 mm/min. To measure deflection during the flexural tests, an MTS video extensometer (AVX-205) was again used. As shown in Figure 8, seven dots were placed on the test specimen. The central dot was positioned at the midpoint of the span. The interval between two consecutive dots was 10 mm. The video extensometer tracked the displacements of these seven dots in both the X and Y-direction throughout testing. Figure 8 also shows a real-life image of the flexural test setup with the seven dots for flax fiber composites.

## 3. Results and Discussion

### 3.1. Tensile Properties of Hemp and Flax Fiber

**Figure 9:**
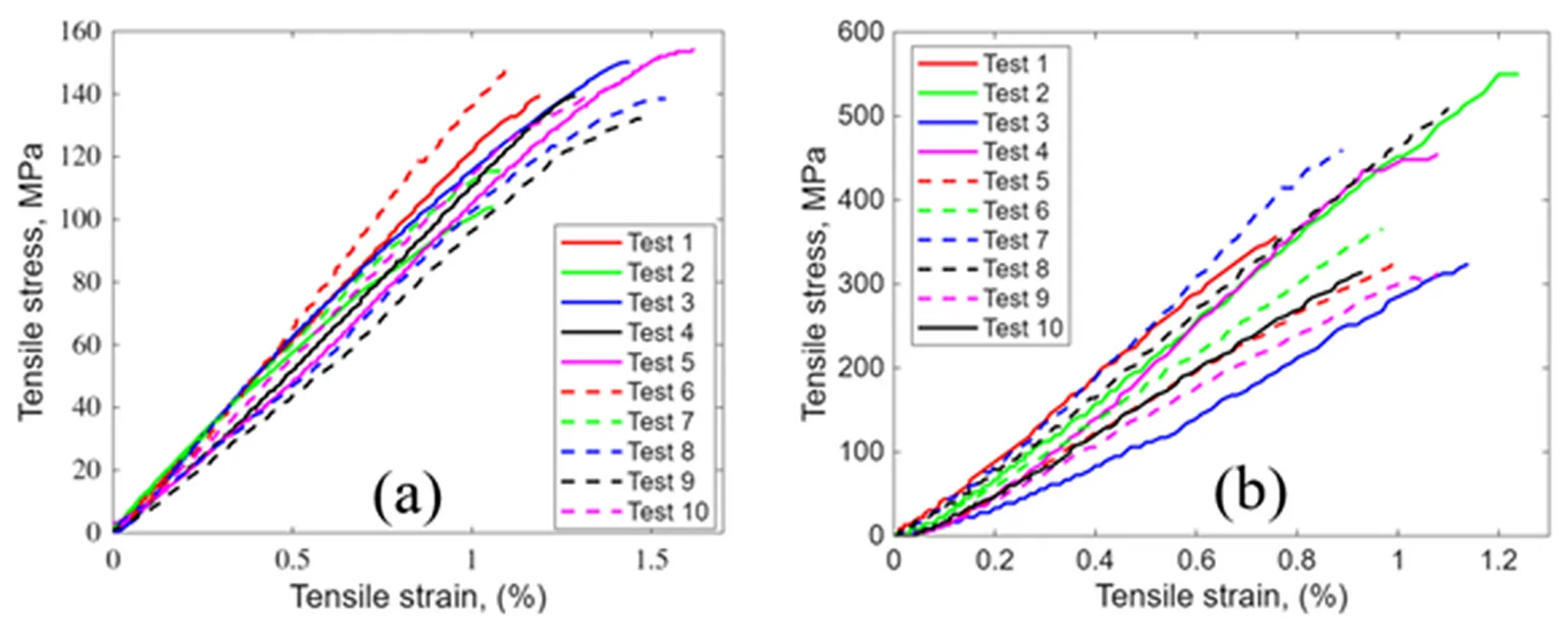
Stress–strain curves from the tensile test of individual fibers: (**a**) hemp and (**b**) flax.

Table 1 shows the tabulated mechanical properties of hemp and flax fibers. The average tensile strength of the individual hemp fibers was 136 ± 15 MPa, the tensile modulus was 11.4 ± 1.8 GPa, and the strain to failure was 1.4 ± 0.2%. In comparison, the flax fibers showed higher values, with an average tensile strength of 397 ± 88 MPa, a tensile modulus of 42 ± 7.1 GPa, and a strain to failure of 1.0 ± 0.13%. Therefore, flax fiber exhibited 190% higher tensile strength and 270% higher tensile modulus compared to hemp fiber. Figure 9a presents the stress–strain curve for hemp fiber, while Figure 9b presents the stress–strain curve for flax fiber. Both fibers exhibited linear stress–strain behavior.

Flax fibers generally exhibit higher tensile properties than hemp fibers due to differences in their intrinsic structural characteristics and chemical composition. Flax fibers typically contain a higher proportion of cellulose and a more uniform arrangement of cellulose microfibrils, which contributes to higher crystallinity, stiffness, and strength. In addition, flax fibers possess a more compact and well-organized internal structure with fewer defects, enabling more efficient load transfer along the fiber axis. Hemp fibers, although also rich in cellulose, generally have higher variability in fiber diameter, greater lumen size, and more heterogeneous structures, which can reduce their effective load-bearing capability. Furthermore, variations in cultivation conditions, retting processes, fiber extraction methods, and moisture content can influence the mechanical properties of both fibers. Therefore, the higher tensile strength observed for flax compared to hemp fibers is attributed to its superior microstructural organization and lower structural variability [24].

Stochioiu et al. [25] reported average tensile strengths of 475 ± 75.49 MPa and 565.12 ± 44 MPa for individual hemp and flax fibers, respectively. In contrast, Sadeghi et al. [26] reported a lower average tensile strength of 97.33 MPa for hemp fiber bundles with varying bundle diameters. The lower strength of fiber bundles compared to individual fibers is primarily attributed to inefficient load transfer among constituent fibers, non-uniform stress distribution, fiber misalignment, and the presence of defects within the bundle. Bundle diameter also influences the measured strength, as larger bundles are more likely to contain weak fibers and structural imperfections that promote premature failure. Furthermore, the tensile properties of natural fibers are known to exhibit considerable variability due to differences in plant species, cultivation conditions, harvesting age, retting and extraction processes, moisture content, gauge length, and testing methodology.

In the present study, the tensile strengths of hemp and flax fibers were determined using twisted fiber bundles rather than individual elementary fibers. Therefore, the measured tensile strengths may not be directly comparable to values reported for single fibers in the literature. The presence of fiber twist introduces additional effects, including changes in fiber alignment, packing density, and stress distribution, which can influence the measured mechanical response. Consequently, the tensile strength values obtained in this study should be interpreted in the context of twisted fiber bundle testing and compared with studies employing similar testing configurations.

### 3.2. Fiber Volume Fraction and Density of 3D Printed Composites

The computed fiber volume fraction for hemp fiber reinforced composites was 13.9%, while the computed fiber volume fraction for flax fiber reinforced composites was 13.5%. Both 3D printed composite specimens had comparable fiber volume fractions. The fiber volume fraction could be further increased by optimizing key printing parameters, such as line spacing, nozzle tip diameter, and printing speed. Moreover, the average composite density of hemp fiber-reinforced composites was measured to be 1490 kg/m^3^, while the average composite density for flax fiber-reinforced composites was 1200 kg/m^3^.

The measured densities of the hemp and flax fiber composites differed by approximately 20%, despite having comparable fiber volume fractions. This difference can be attributed to the intrinsic density variation between hemp and flax fibers, as well as differences in composite morphology. Although the fiber volume fractions are similar, the mass-based density of the composite is strongly influenced by the constituent material densities. Flax fibers generally possess a slightly higher density than hemp fibers due to differences in cellulose content, lignin composition, and fiber microstructure, which can contribute to the higher overall density of flax fiber composites. Additionally, variations in fiber packing, bundle structure, resin distribution, and void content can affect the measured composite density. Therefore, similar fiber volume fractions do not necessarily result in identical composite densities, as the density measurement reflects the combined influence of fiber properties, matrix content, and internal structural characteristics.

### 3.3. Surface Analysis of Printed Layer

**Figure 10:**
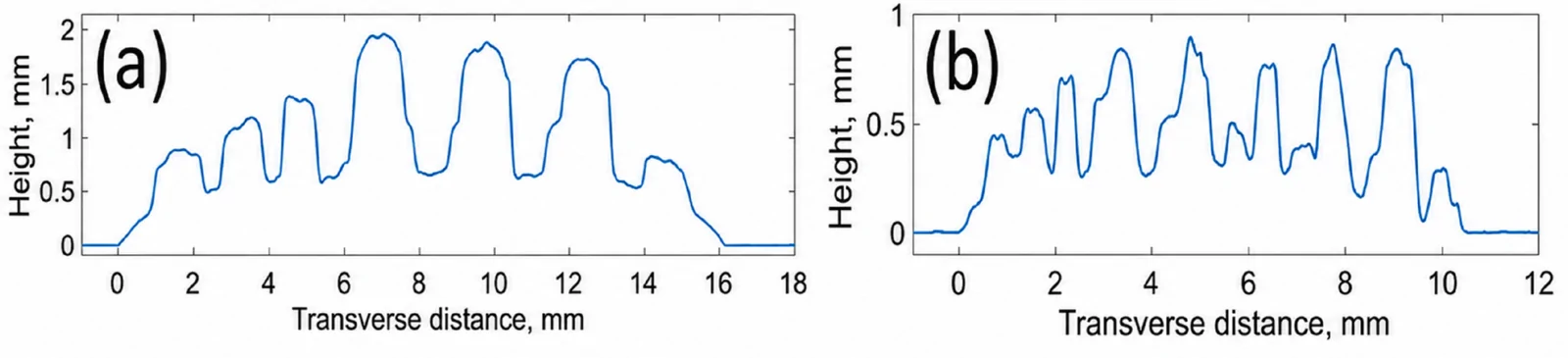
Surface profile of the printed layer: (**a**) hemp composites; (**b**) flax composites.

The measured average line roughness was 216.06 ± 5.16 μm for hemp fiber-reinforced composites and 136 ± 14.04 μm for flax fiber-reinforced composites. Compared to the surface of 3D-printed hemp fiber-reinforced composites, the line roughness of 3D-printed flax fiber-reinforced specimens was 35% lower, resulting in a smoother surface. Additionally, Figure 10a shows the surface profile of the 3D printed single layer along the transverse direction for hemp fiber-reinforced composites, while Figure 10b shows the profile for flax fiber-reinforced composites.

The difference in surface roughness between hemp and flax fiber-reinforced composites may influence their mechanical performance by affecting the fiber–matrix interfacial bonding. A rougher fiber surface can enhance mechanical interlocking and increase the effective contact area with the resin, potentially improving stress transfer between the fiber and matrix. Therefore, the variation in mechanical properties between hemp- and flax-reinforced composites may be partially attributed to differences in their surface morphology and the resulting interfacial adhesion.

### 3.4. Tensile Properties of Printed Composites

**Figure 11:**
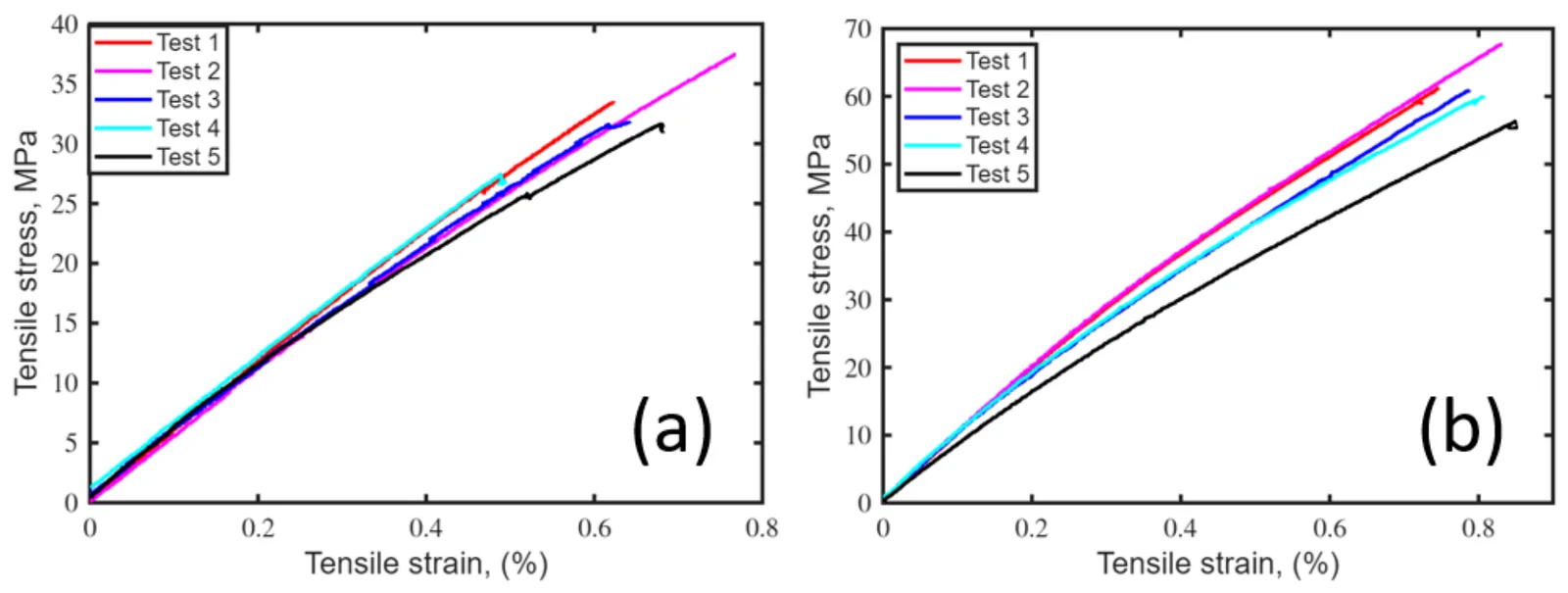
Stress–strain curve of tensile test for (**a**) hemp fiber composites and (**b**) flax fiber composites.

Table 2 shows the tabulated mechanical properties of hemp and flax fiber-reinforced 3D-printed composites. Figure 11a presents the tensile stress–strain curves of the 3D-printed continuous hemp fiber-reinforced thermoset composites, while Figure 11b shows the tensile stress–strain curves of the flax fiber-reinforced 3D-printed composites. The hemp fiber-reinforced composites exhibited an average tensile strength of 32.3 ± 3.5 MPa and an average tensile modulus of 5.7 ± 0.19 GPa. In comparison, the flax fiber-reinforced composites achieved an average tensile strength of 61.1 ± 4.2 MPa and a tensile modulus of 9.6 ± 0.75 GPa. Therefore, the flax fiber-reinforced composites demonstrated approximately 90% higher tensile strength and 70% higher tensile modulus than the hemp fiber-reinforced composites.

The tensile properties obtained in this study are comparable with previously reported natural fiber-reinforced thermoset composites. Fernández et al. [27] investigated short hemp fiber-reinforced unsaturated polyester composites with the addition of 5 wt% bentonite nanoparticles and reported a maximum tensile strength of 34.28 MPa for the composite containing 35 wt% hemp fibers. The composites were manufactured using a conventional fabrication method with box-type molds. The continuous hemp fiber-reinforced composite developed in the present study achieved a comparable tensile strength (32.3 MPa) without nanoparticle modification. This performance can be attributed to the continuous fiber architecture, which promotes efficient load transfer through improved fiber alignment and reduced fiber discontinuity. Moreover, differences in fiber architecture, matrix properties, fiber content, and fabrication methods contribute to variations in reported mechanical performance.

Similarly, Bhadana et al. [28] reported a tensile strength of 62 MPa for flax/epoxy composites developed for structural applications. The flax fiber-reinforced composite fabricated in the present study exhibited a comparable tensile strength (61.1 MPa), demonstrating the capability of the additive manufacturing approach to achieve mechanical performance similar to conventionally manufactured continuous flax fiber composites. However, variations in fiber volume fraction, impregnation quality, void content, and processing conditions can influence the measured tensile properties. The superior tensile performance of flax fiber-reinforced composites compared with hemp fiber-reinforced composites is consistent with previous studies. Higher tensile properties of the 3D printed flax fiber composites are more likely linked with the higher tensile properties of the flax fiber.

The stress–strain curve exhibited non-linearity for both hemp and flax fiber-reinforced composites. This phenomenon has also been reported in the literature for conventionally manufactured flax fiber-reinforced composites [29]. This behavior can be explained by internal structural changes in natural fibers.

**Figure 12:**
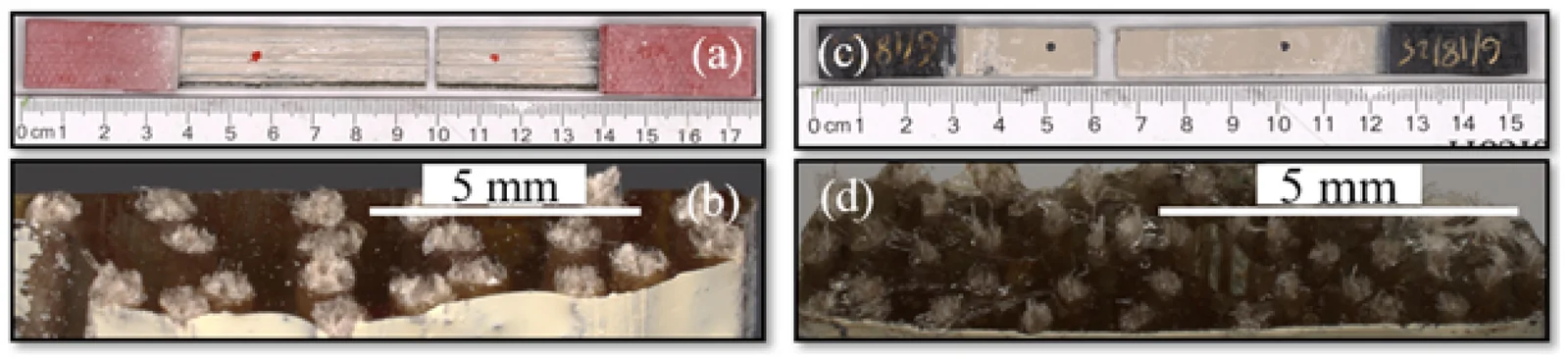
Tensile tested specimen and fracture surface under optical microscope for (**a**,**b**) hemp fiber composites and (**c**,**d**) flax fiber composites.

A failed tensile specimen for hemp fiber composites is shown in Figure 12a, while Figure 12b presents the fracture surface image captured under an optical microscope (Keyence, VHX-6000). However, A failed tensile specimen for flax fiber composites is shown in Figure 12c, while Figure 12d presents the fracture surface image captured under an optical microscope. Both fracture surfaces of hemp and flax composites show evidence of fiber tow pull-out. Furthermore, the matrix material exhibits a cleaner fracture in the case of hemp composites, compared to flax fiber composites. The failure mechanism was more brittle in nature in the case of hemp fiber-reinforced composites. Figure 12b,d further shows evidence of improper resin impregnation within the fiber tow. Due to the smaller size of the tow, flax fiber tow in Figure 12d shows better resin impregnation compared to the hemp fiber composites. This poor impregnation is attributed to the use of thicker, twisted fiber tows of hemp fiber. Utilizing thinner fiber tows may improve resin penetration and result in enhanced tensile properties.

**Figure 13:**
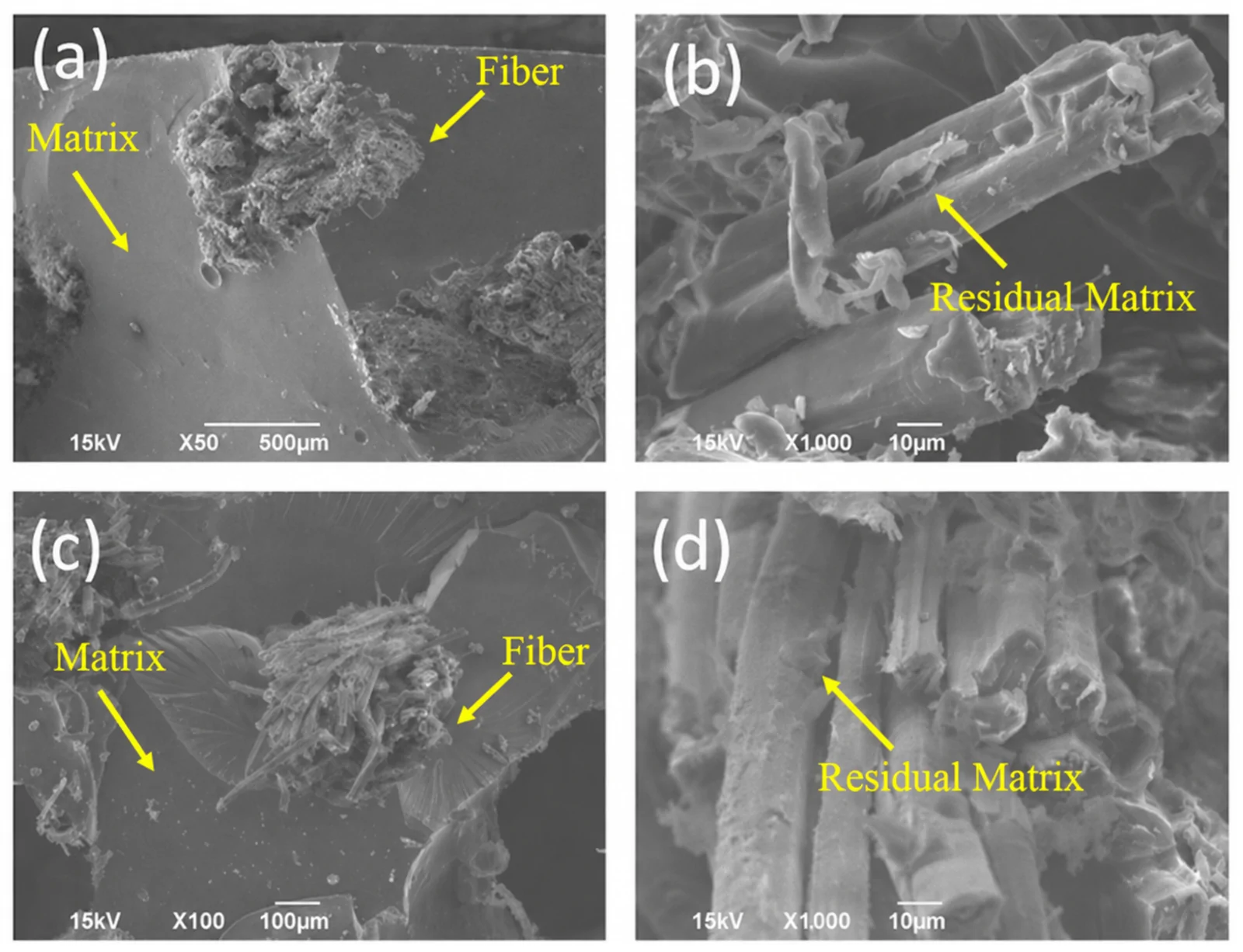
Fracture surface under SEM for (**a**,**b**) hemp composites and (**c**,**d**) flax composites.

Additionally, Figure 13a,b presents SEM images of the fracture surfaces of hemp fiber-reinforced composites, whereas Figure 13c,d shows the fracture surfaces of flax fiber-reinforced composites. The SEM characterization was conducted using a JEOL JSM-35 scanning electron microscope (Tokyo, Japan). The hemp composites exhibit relatively clean and exposed fiber surfaces with limited residual matrix, indicating weaker fiber–matrix adhesion and greater interfacial debonding and fiber pull-out during fracture. In contrast, the flax composites show a greater amount of residual matrix adhered to the fiber surfaces, suggesting better resin impregnation and stronger mechanical interlocking. These observations are consistent with the higher tensile performance of the flax composites, as stronger interfacial bonding facilitates more effective stress transfer from the matrix to the fibers.

The difference in fiber diameter may also contribute to the observed interfacial behavior. The flax fibers used in this study were thinner than the hemp fibers, which can provide greater surface area relative to fiber cross-sectional area and facilitate resin penetration around the fibers during printing. Consequently, the thicker hemp fibers may have experienced comparatively less effective resin impregnation, increasing the likelihood of interfacial defects and fiber pull-out. Furthermore, the flax fibers possessed higher intrinsic tensile strength than the hemp fibers used in this study. Therefore, the combination of better fiber–matrix interaction and higher fiber strength contributed to the higher tensile strength of the flax fiber-reinforced composites compared with the hemp fiber-reinforced composites. The observed fracture features thus demonstrate that both fiber strength and interfacial bonding/impregnation play important roles in determining the tensile performance of the printed natural-fiber composites.

### 3.5. Flexural Properties of Printed Composites

The hemp fiber-reinforced composites exhibited an average flexural strength of 81.8 MPa with a standard deviation of 13.5 MPa, and an average flexural modulus of 6 ± 0.8 GPa. In comparison, the flax fiber-reinforced composites showed an average flexural strength of 85.3 MPa with a standard deviation of 23.1 MPa, and an average flexural modulus of 7.6 ± 2.6 GPa. Hence, the 3D printed flax fiber-reinforced composites showed 4.3% higher flexural strength and 27.3% higher flexural modulus compared to the 3D printed hemp fiber-reinforced composites. Although the 3D-printed thermoset composites demonstrated lower flexural properties than thermoplastic counterparts reported in the literature [15], this preliminary study lays the groundwork for UV-light-assisted 3D printing of natural fiber composites.

**Figure 14:**
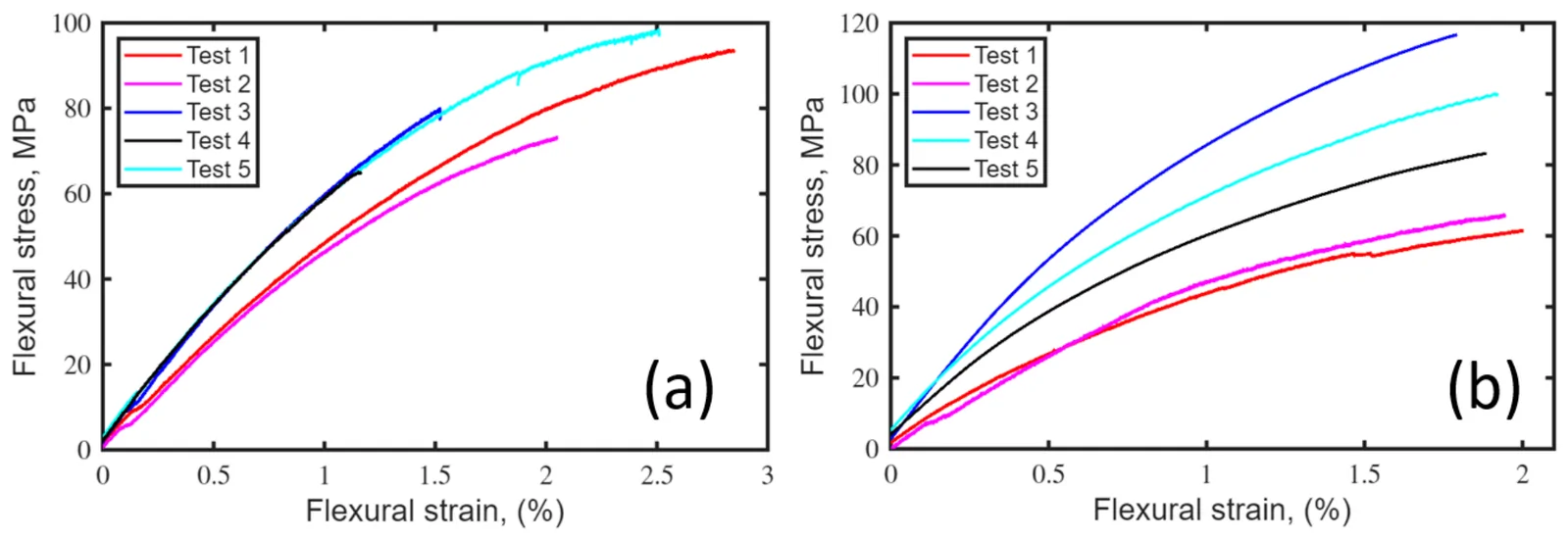
Stress vs. strain curves for flexural test: (**a**) for hemp composites, (**b**) for flax composites.

Figure 14 shows the flexural test curves for both hemp and flax fiber reinforced 3D printed composites. During the test, the average flexural strain at failure for hemp fiber composites was 2.4%, while the average flexural strain for flax fiber-reinforced composites was 1.9%. Therefore, the 3D printed specimens exhibited significant deflection before failure.

**Figure 15:**
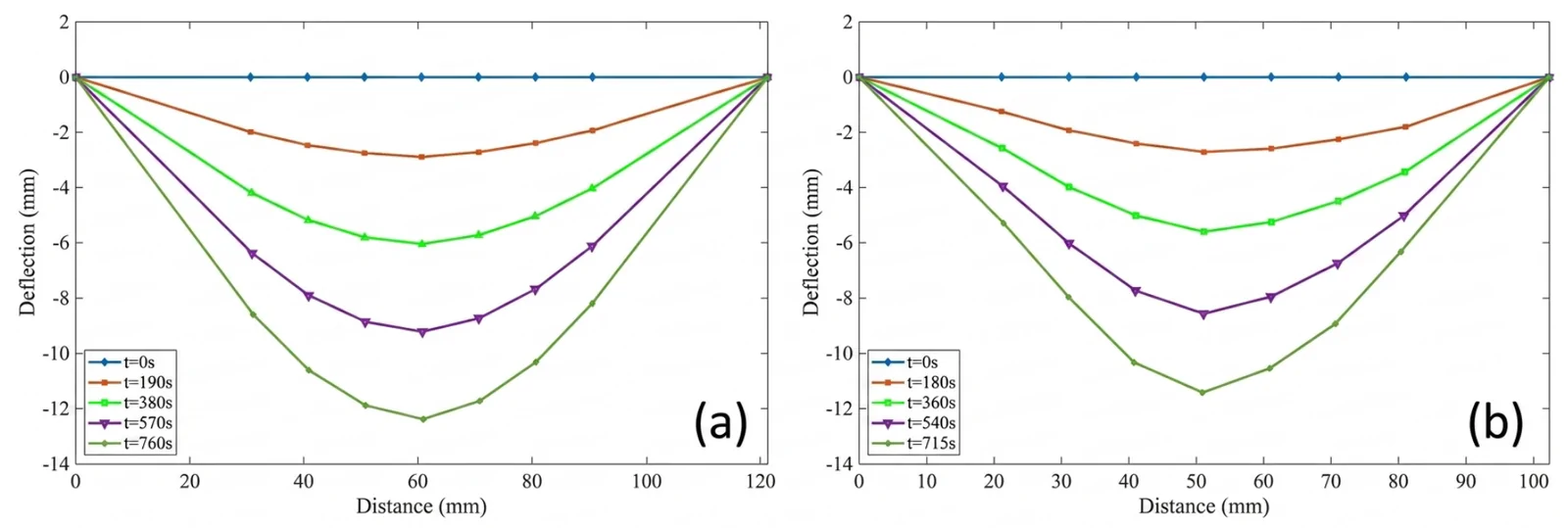
Deflection curve of flexural test specimen at various times: (**a**) hemp fiber composites; (**b**) flax fiber composites.

Figure 15a shows the deflection curves of hemp fiber-reinforced composites during flexural testing at different time intervals, while Figure 15b presents the corresponding curves for flax fiber-reinforced composites. As mentioned earlier, the deflection was captured using the video extensometer. The total test duration was approximately 760 s for hemp fiber-reinforced composites and 715 s for flax fiber-reinforced composites. The deflection curves for hemp fiber-reinforced composites are shown at 0, 190, 380, 570, and 760 s, while those for flax fiber-reinforced composites are shown at 0, 180, 360, 540, and 715 s. The total vertical deflection before failure was 12.4 mm for hemp fiber-reinforced composites. The total vertical deflection before failure was 11.4 mm for flax fiber-reinforced composites. Thus, both composite specimens deformed significantly before failure.

**Figure 16:**
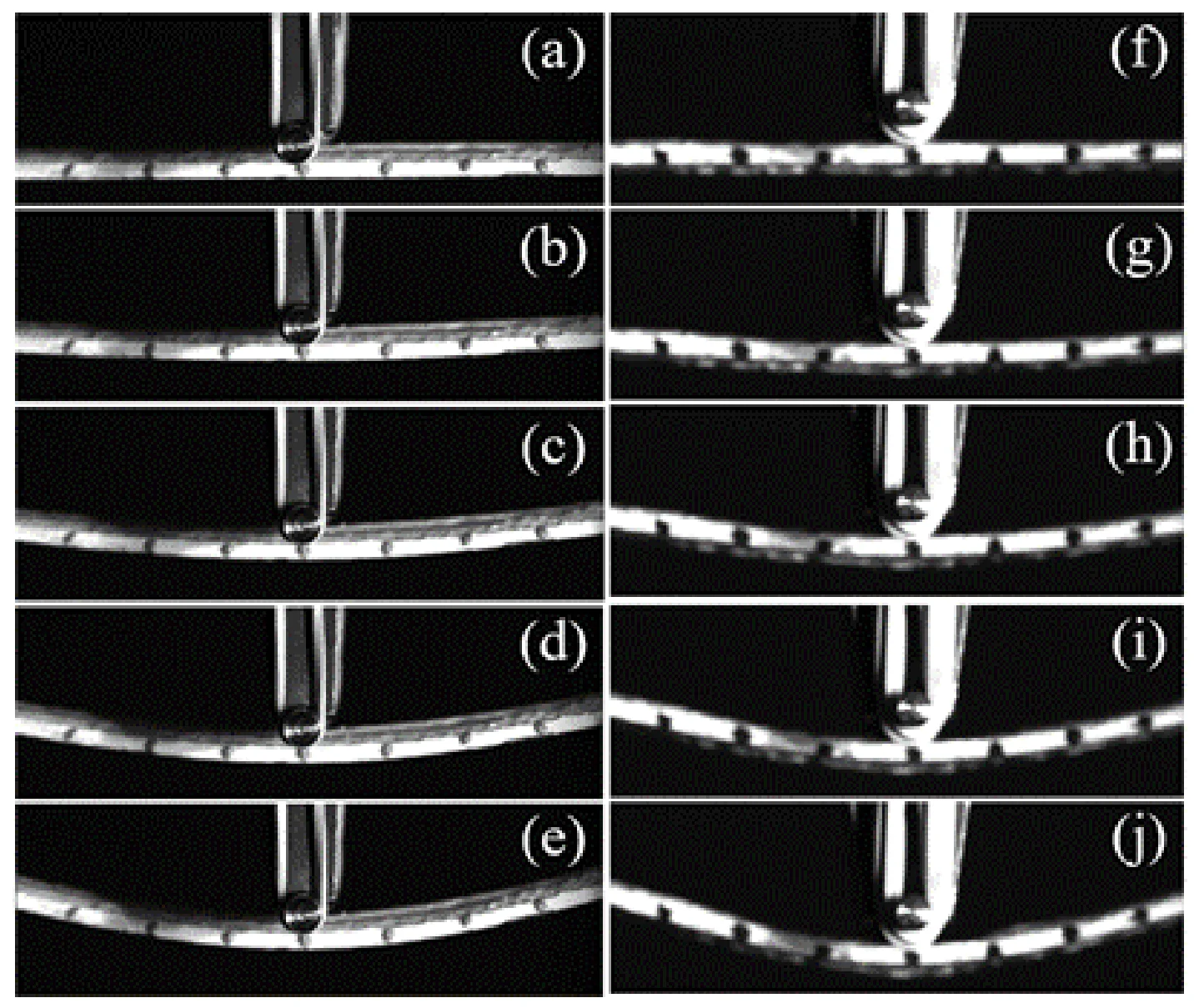
Captured deflection images of flexural test specimen at various times: (**a**–**e**) hemp fiber specimen at 0, 190, 380, 570, 760 s, respectively; (**f**–**j**) flax fiber specimen at 0, 180, 360, 540, 715 s, respectively.

Additionally, the deflection images of the specimen during the flexural test at various times are shown in Figure 16. Figure 16a–e shows the deflection images for the hemp fiber composites, while Figure 16f–j shows the deflection images for the flax fiber composites. These images were captured using the MTS video extensometer. The video extensometer’s frame only captured the seven dots instead of the full span. However, these images provide qualitative information about the complete deflection pattern of the 3D-printed continuous fiber-reinforced composite. Both composites exhibit smooth and regular deflection curves, indicating stable deformation behavior under flexural loading. Flax fiber composites exhibit a higher flexural modulus and therefore demonstrate lower deflection under flexural loading.

## 4. Conclusions

This study successfully demonstrated, for the first time, the full process of 3D printing hemp and flax fiber-reinforced thermoset composites using a light-assisted process. The printed specimens achieved fiber volume fractions of 13.9% (hemp) and 13.5% (flax). As this work is a preliminary demonstration of the process, future research will focus on optimizing manufacturing parameters, such as fiber handling, resin impregnation, nozzle design, and deposition conditions, to increase the fiber volume fraction and further improve the mechanical performance of the printed composites. The difference in fiber diameter and tensile strength between the two fiber types was found to influence the resulting composite performance. The thinner flax fibers facilitated comparatively better resin impregnation and fiber–matrix interaction, while their higher intrinsic tensile strength contributed to the higher tensile strength of the flax composites. SEM observations further indicated greater fiber pull-out and interfacial debonding in the hemp composites, whereas the greater residual matrix observed on the flax fibers suggested stronger fiber–matrix adhesion and more effective stress transfer. Mechanical testing showed that flax composites exhibited higher tensile strength (61.1 ± 4.2 MPa) compared to hemp composites (32.3 ± 3.5 MPa), while both materials achieved comparable flexural strengths of approximately 81–85 MPa with significant deformation prior to failure. These results demonstrate that tensile performance was more strongly influenced by fiber strength and fiber–matrix interaction, whereas the comparable flexural performance indicates that the two fiber systems can provide similar load-bearing capability under bending despite their differences in tensile behavior. These findings confirm the capability of integrating continuous natural fibers into additively manufactured thermoset composites and open new research pathways to produce lightweight, high-performance, and biodegradable materials for sustainable engineering applications. Further research could focus on optimizing fiber–matrix interactions and studying the interlaminar bonding strength, impact and fatigue behavior of the composites to validate their real-life applicability.

## 5. Future Outlook

The successful integration of continuous hemp and flax fibers into additively manufactured thermoset composites provides opportunities for lightweight and sustainable components in applications where moderate mechanical performance is required. Compared with conventional manufacturing methods, additive manufacturing can reduce material waste by depositing material only where it is needed, while also enabling complex geometries and part consolidation that may be difficult or costly to achieve using traditional processes. In addition, the ability to manufacture near-net-shape components directly from digital designs can reduce tooling requirements, shorten production lead times, and support cost-effective customization and rapid design iteration. Future studies should focus on increasing fiber volume fraction, improving resin impregnation and fiber–matrix bonding, and evaluating long-term durability, moisture resistance, impact, fatigue, and interlaminar properties. These developments could enable the use of continuous natural-fiber thermoset composites in automotive, consumer, and other lightweight engineering applications while improving the sustainability of additively manufactured composite components.

## Figures and Tables

**Table 1:** Mechanical properties of hemp and flax fibers.

	Hemp	Flax
Tensile Strength (MPa)	136 ± 15	397 ± 88
Tensile Modulus (GPa)	11.4 ± 1.8	42 ± 7.1
Strain-to-failure (%)	1.4 ± 0.2	1.0 ± 0.13

**Table 2:** Mechanical properties of 3D-printed composites reinforced with hemp and flax fibers.

	Hemp	Flax
Volume Fraction (%)	13.9	13.5
Density (kg/m^3^)	1490	1200
Line Roughness (μm)	216.06 ± 5.16	136 ± 14.04
Tensile Strength (MPa)	32.3 ± 3.5	61.1 ± 4.2
Tensile Modulus (GPa)	5.7 ± 0.2	9.6 ± 0.8
Flexural Strength (MPa)	81.8 ± 13.5	85.3 ± 23.1
Flexural Modulus (GPa)	6.0 ± 0.8	7.6 ± 2.6

## Data Availability

The original contributions presented in this study are included in the article. Further inquiries can be directed to the corresponding author.
